# Biological and environmental drivers of trophic ecology in marine fishes - a global perspective

**DOI:** 10.1038/s41598-019-47618-2

**Published:** 2019-08-06

**Authors:** B. Hayden, M. L. D. Palomares, B. E. Smith, J. H. Poelen

**Affiliations:** 1 Canadian Rivers Institute, Biology Department, University of New Brunswick, Fredericton, New Brunswick Canada; 2 Sea Around Us, University of British Columbia, Vancouver, Canada; 3 Quantitative Aquatics, Los Baños, Laguna, Philippines; 4 NOAA Fisheries, Northeast Fisheries Science Center, 166 Water Street, Woods Hole, Massachusetts, 02543 USA; 5 Freelance Developer, Oakland, CA 94610 USA

**Keywords:** Fisheries, Ichthyology, Ecology, Biodiversity, Marine biology

## Abstract

Dietary niche width and trophic position are key functional traits describing a consumer’s trophic ecology and the role it plays in a community. However, our understanding of the environmental and biological drivers of both traits is predominantly derived from theory or geographically restricted studies and lacks a broad empirical evaluation. We calculated the dietary niche width and trophic position of 2,938 marine fishes and examined the relationship of both traits with species’ maximum length and geographic range, in addition to species richness, productivity, seasonality and water temperature within their geographic range. We used Generalized Additive Models to assess these relationships across seven distinct marine habitat types. Fishes in reef associated habitats typically had a smaller dietary niche width and foraged at a lower trophic position than those in pelagic or demersal regions. Species richness was negatively related to dietary niche width in each habitat. Species range and maximum length both displayed positive associations with dietary niche width. Trophic position was primarily related to species maximum length but also displayed a non-linear relationship with dietary niche width, whereby species of an intermediate trophic position (3–4) had a higher dietary niche width than obligate herbivores or piscivores. Our results indicate that trophic ecology of fishes is driven by several interlinked factors. Although size is a strong predictor of trophic position and the diversity of preys a species can consume, dietary niche width of fishes is also related to prey and competitor richness suggesting that, at a local level, consumer trophic ecology is determined by a trade-off between environmental drivers and biological traits.

## Introduction

The concept of a strict ecological niche to which a species is confined has been challenged on multiple occasions but is currently enjoying a revival as ecologists use species’ functional traits to describe their role within ecological networks, their response to environmental change and their contribution to ecosystem function^1,2^. The ecological niche was first formalized by Grinnell who demonstrated that a species’ morphology and behavior is adapted to its environment^3^. The Grinnelian Niche was later advanced by Elton to include a species relationship with its predators and prey^4^. Hutchinson subsequently portrayed the niche as an n-dimensional hypervolume, whereby species may be classed as specialist or generalist in accordance with their performance under a narrow or wide suite of environmental conditions^2^. The dietary niche of a consumer, i.e., the diversity of prey types eaten by an individual, population or species is an integral component of its ecological niche^5^. Despite its importance in ecology, the factors which determine whether a species will occupy a small (i.e., dietary specialist) or a large dietary niche (i.e., dietary generalist) remain unclear. Classic niche theory indicates that dietary generalists will be prevalent in regions where prey is scarce and competitors are few, but that highly bio-diverse regions will contain a greater number of dietary specialists, as potential prey and competitors are found in abundance^6^. However, diet is also determined by a host of environmental and biological traits. For example, fishes adapted to benthic or pelagic habitats will be exposed to entirely different prey communities; a predator’s morphology, especially its size, mobility and dentition will determine what prey types it can capture; and the geographic range of a consumer will also influence the breadth of prey it may consume^7^. Furthermore, a species’ trophic position, i.e., their position on a food chain, may be non-linearly related to its dietary niche width, as omnivores consume a greater diversity of prey types than either herbivores or carnivores.

Trophic position, in turn, is also influenced by resource availability; in regions of low resource availability, consumers are likely to forage across multiple trophic levels whereas, the high diversity of prey and competitors in biodiversity hotspots may allow consumers to specialize at a single trophic position^7^. Thus, characterizing the dietary niche width and trophic position of consumers across global geographic and richness scales may bridge the gap between the trophic ecology of consumers and their role in ecosystem function.

Assessing these relationships at a global scale using observational rather than theoretical approaches presents numerous challenges, and until recently was infeasible. However, the development of open-access data archives compiling the functional and life history traits of fishes^8^ in addition to global quantification of marine species richness^9^ provide a unique opportunity to identify global correlates of species’ dietary niche width and trophic position^10^. Here, we first determine the dietary niche width (DNW) and trophic position (TP) of 2,938 marine fishes and subsequently use Generalized Additive Models (GAM) to examine factors influencing both traits. We tested four hypotheses related to the environmental and biological traits which underpin a consumer’s trophic ecology: (1) DNW and TP are negatively related to prey and competitor richness^7^; (2) DNW and TP are positively related to species maximum length as larger species can feed on a greater variety of prey (i.e., small and large prey items) and therefore may occupy a higher trophic position^11^; (3) DNW and TP are positively related to species range size as species with a broader geographic distribution are likely to be exposed to a greater diversity of prey^12^; (4) Species with an intermediate trophic position (i.e. 3–3.5) have a higher DNW than either specialist herbivores or piscivores^10^.

## Methods

### Data acquisition

#### Dietary niche width (DNW)

We combined fish diet data from FishBase (ww.fishbase.org) with other published datasets (list of data sources in Supporting Table 1) using the Global Biotic Interactions database^13^. Where a fish species was found in more than one dataset, all records were pooled to create a ‘total observed diet’ for that species across multiple sizes, life stages, locations, habitats and years. As the classification level and terminology of prey items varied between datasets, we assigned items recorded in the diet to 29 pre-determined prey categories (see Supporting Table 2). Prey categories were defined at Class level to ensure that DNW reflected the utilization of distinct prey types (e.g., fishes and gastropods) rather than foraging in an area of high taxonomic richness (e.g., multiple species of gastropod). Approximately 10% (970 of 11,605) of prey items could not be matched to any category (e.g., unidentified material) and were omitted from further analysis.

The DNW of each species was calculated as the ratio of the number of prey categories recorded in a species’ diet relative to the total number of potential prey types:

$$DNW=\frac{\sum (P1+P2\ldots \,.Px)}{Np}$$

where *P* is a binary presence/absence score for each prey category and *Np* is the number of potential prey categories, i.e. 29^5^.

This measure of dietary niche width is cumulative and as such may be biased by the amount of data available for each particular species. To account for this potential bias in our data we used the number of observations of each species in the Global Biodiversity Information Facility (GBIF) database^14^ as a proxy for the amount of data available for each species, assuming that the relative number of occurrences in GBIF reflects the relative availability of dietary records for each species.

#### Trophic position (TP)

The most common method of estimating trophic position is a binary approach that indicates the presence or absence of feeding links and is usually represented as an integer^15^. This has been criticized as failing to properly account for omnivory^16^, otogenetic and spatial variation^17^. The trophic position of each consumer species in this study was obtained from FishBase^8^, which estimates trophic position as: TP_i_ = 1 + ∑_j_ TP_j_*DC_ij_, where TP_i_ is the fractional trophic position of the preys j, and DC_ij_ represents the fraction of j in the diet of i^18^. This defines the trophic position of most consumers between 2.0 and 5.0, i.e., as measurable fractional entities that may be cross-validated using different methods, e.g., mass balance models of trophic fluxes^19^ or ratios of ^15^N to ^14^N^20^. Note that for fishes, even for large species, we do not expect trophic positions reaching the maximum of 5.0^21^, which is rare and occurs only in killer whales and polar bears^22^. Trophic position values were extracted from FishBase using the rFishBase package (ver 5/2017)^23^.

#### Species richness

We calculated species richness using data available in AquaMaps^24^. AquaMaps aggregates data from GBIF, OBIS and other sources to generate a list of all species identified in 259,201 grid-squares (each 2,700 km^2^) encompassing the Earth’s oceans and currently contains over 30 million records of species * location interactions. We used the number of species reported in each grid-square as a proxy for overall species richness in that grid-square. The mean species richness of all grid-squares in which a consumer species was recorded, was used as a proxy for species richness within that consumer’s range (hereafter richness). The richness metric was log transformed to reduce skew towards extremely species rich grid-squares.

#### Species range and habitat

The geographic range size of each consumer species was inferred from the number of AquaMaps grid-squares in which it had a 90% or greater probability of occurring. These values were log transformed prior to analysis to reduce a skew towards small ranges. The habitat of each species was obtained from the ‘*DemersPelag*’ category in FishBase, species were assigned to one of seven primary marine habitats: Bathydemersal (living and feeding on the sea bed at depths below 200 m), Bathypelagic (living and feeding in open water depths below 200 m), Benthopelagic (foraging across benthic and pelagic habitats), Demersal (living and feeding near the sea bed shallower than 200 m and not reef-associated), Pelagic-neritic (living and feeding in the pelagic zone above a continental shelf), Pelagic-oceanic (living and feeding in the pelagic zone of the open ocean) and Reef-associated (living and feeding on a wave resistant feature the upper surface of which is within 0–20 m of the ocean surface). The principal habitat of each species was obtained using rFishBase (ver 5/2017)^23^.

#### Species length

Max length of each species was obtained from FishBase^23^. FishBase predominantly records max length as either standard length (snout to posterior end of the last vertebrae) or total length (snout to tip of the longer lobe of the caudal fin). As only one type of measurement was available for each species we were forced to include an assumption that this level difference in length would not be sufficient to bias our analysis (a comparison of model fits for all fish TP, n = 1881, and SL, n = 679, fish which supports this assumption is presented in Supporting Fig. S1). Due to a distribution skewed towards small sized fishes, species length data were log transformed prior to analysis. Species length values were obtained using rFishBase (ver 5/2017)^23^.

#### Environmental predictors: Sea surface temperature, latitude & productivity

Mean annual (1982–1999) sea surface temperature (SST) at the center-point of all AquaMap grid-squares, derived from the NOAA National Center for Environmental Prediction database, was obtained via AquaMaps. Seasonality has been associated with the resource use of fishes, whereby fishes in regions with high seasonal variability may be expected to forage on wider range of prey types^25^. The mean, annual SST range in each grid-square was obtained from the same dataset and represents a proxy for seasonality within that grid-square. Annual primary production in each grid-square (mgC m^−2^ day^−1^) was obtained from AquaMaps. The mean SST, SST range and productivity values of all grid-squares in which a consumer species had a 90% or higher probability of occurring was used as a proxy for those measurements within the range of that species.

### Data analysis

We used Generalized Additive Models (GAM)^26^ to test the relationships between DNW and TP of each fish species the predictor variables outlined above. GAMs were preferred to generalized linear models as we expected non-linear relationships between variables, e.g., we predicted TP would display a non-linear relationship with DNW. The number of occurrences of each species in GBIF was included in all models to account for effect of unbalanced data availability. We included ‘habitat; as a fixed effect in each model to determine the degree to which trends differed between habitats. Sea surface temperature was highly collinear with species richness (lm: df = 2672, r^2^ = 0.55, P < 0.001, Supporting Fig. S2) and was omitted from models. Collinearity among the remaining variables was within acceptable levels (VIF scores < 1.2), although there was a positive relationship between trophic position and log max length in larger fishes (Fig. S2). All models used a Gaussian distribution and identity link function, smoothing parameters were estimated using GCV^27^, and were simplified with the *select* function which effectively removes unnecessary smoothers from the model. Models were conducted in R *ver*. 3.2.2^28^.

## Results

### Dietary niche width

**Figure 1:**
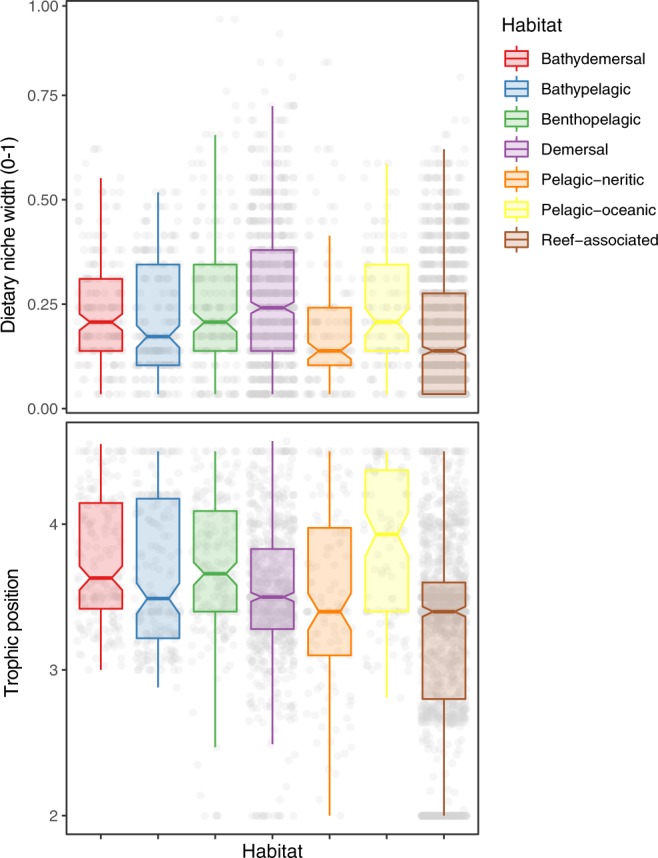
Variation in the dietary niche width and trophic position of fishes in seven major marine habitats. Each point in represents the dietary niche width or trophic position of a species, boxplots detail the median, interquartile range and twice the interquartile range of species associated with each habitat.

**Figure 2:**
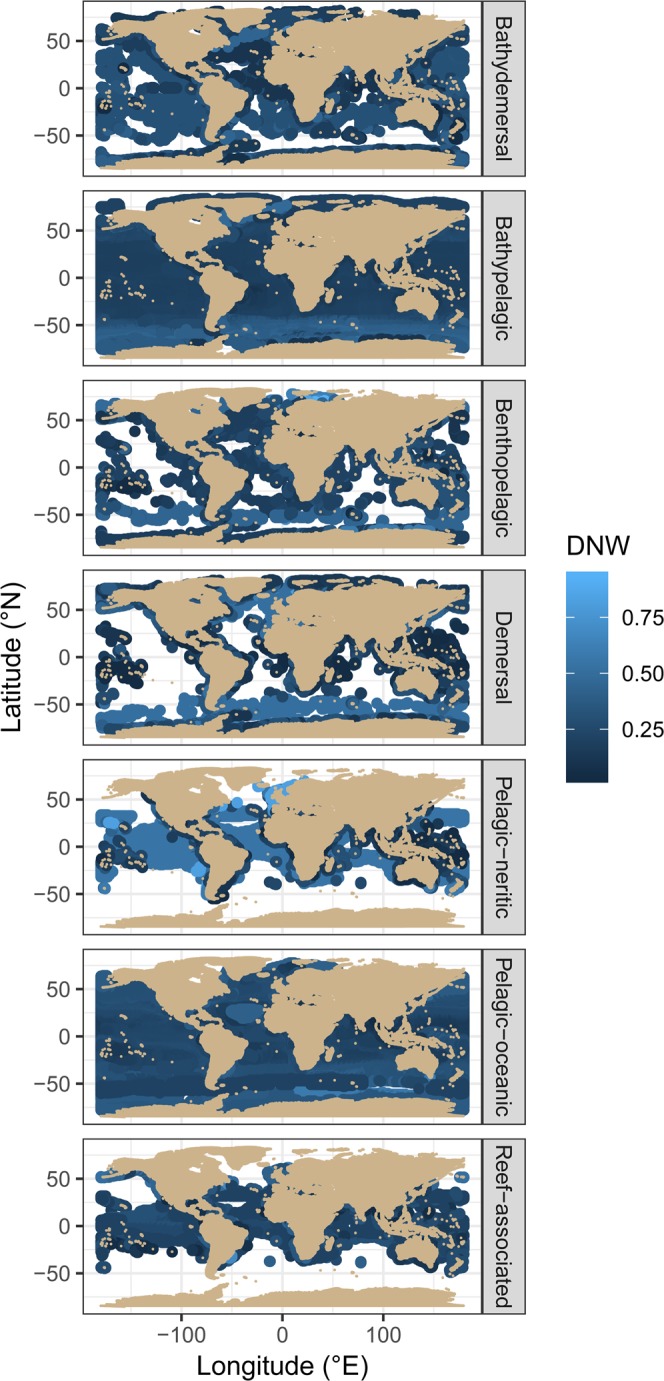
Global variation in the dietary niche width of marine fishes. Each point represents the mean niche with of all fish species with a 90% probability of occurring in that location. Species distribution was estimated from AquaMaps and habitat preference is obtained from FishBase.

Mean DNW across all fish was 0.21 (SD: ±0.16, interquartile range 0.07–0.31), which equates to feeding on approximately 6 of the 29 potential prey categories (interquartile range: 2–9). The range of DNW was relatively consistent across all habitat types (Fig. 1), though pairwise t-tests of the DNW values indicate that reef associated fishes typically have a smaller DNW than demersal species (Table S3). Latitudinal variation in the mean DNW of species was evident across habitats whereby grid-squares close to the equator contained fishes with a smaller dietary niche width than those in Artic or Antarctic seas (Fig. 2).

**Figure 3:**
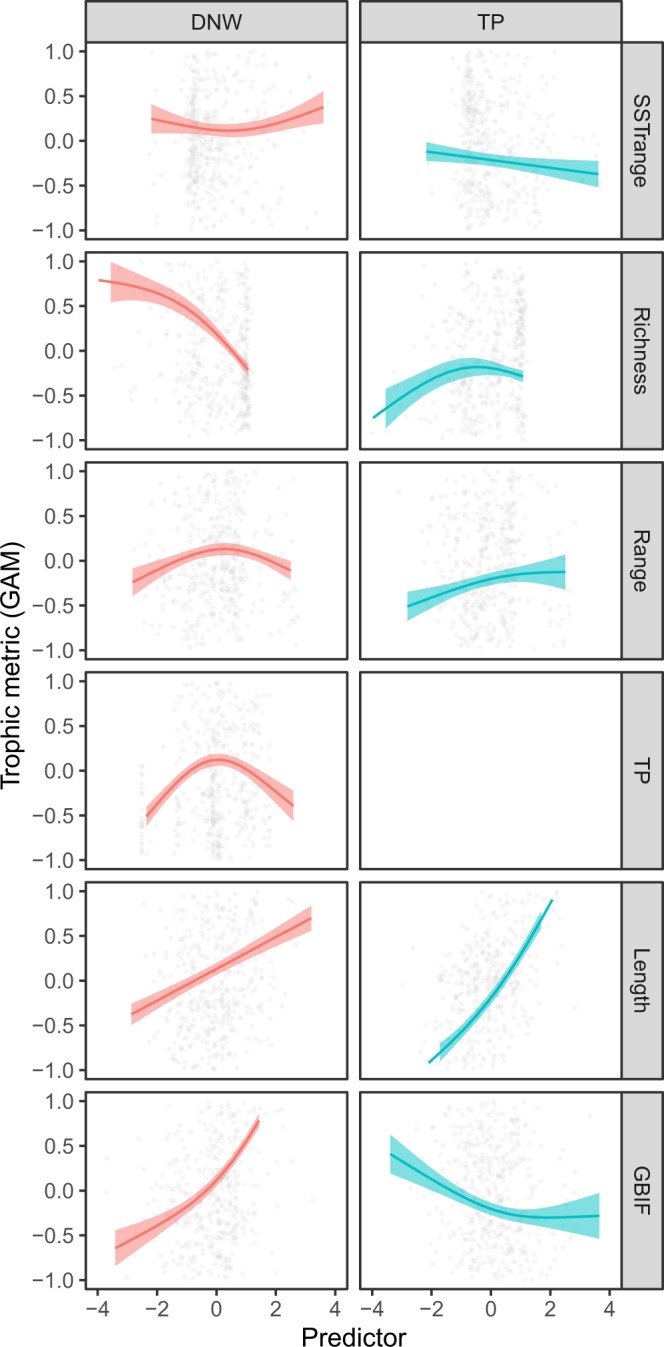
The effect of variation in predicted drives on the dietary niche width (DNW; left panels) and trophic position (TP; right panels) of 2,938 marine fishes. Plots represent relationships indicated by the best fitting GAM (see Table 1). Smoothed functions presented as solid lines, blue shading denotes 2 standard errors, and grey circles indicate each species.

GAM’s indicated a strong positive relationship between a species’ DNW and the number of occurrences of that species in the GBIF database, indicating that the DNW metric is influenced by the amount of data available for a specific species (Table 1, Fig. 3). This trend was evident across all habitats (Table 1, Supporting Fig. S3). The marine productivity within a species’ geographic range had a small but statistically significant positive relationship with the species’ DNW. The data also revealed a non-linear relationship between DNW and the latitude of the mid-point of a species range, whereby species found primarily in equatorial regions has a smaller DNW than those found in polar seas. Further variation in species’ DNW are described below in the context of the four hypotheses set out in the introduction.

### Trophic position

**Figure 4:**
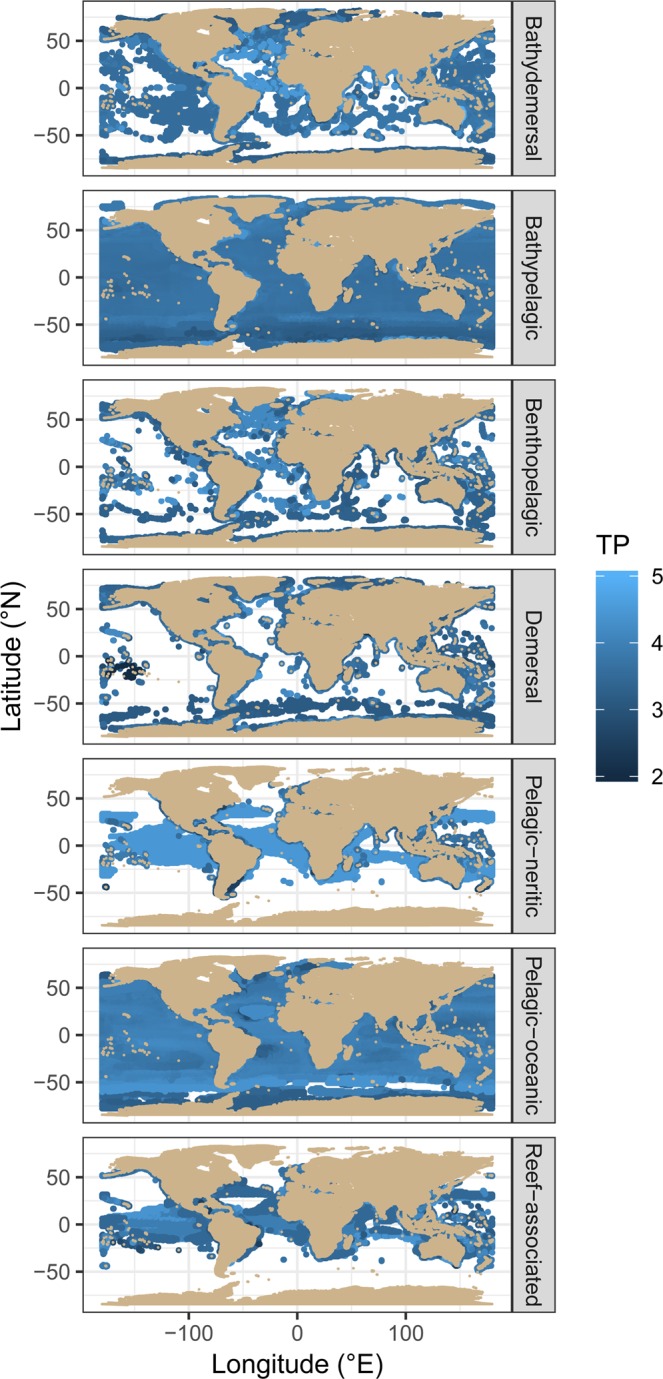
Global variation in the trophic position of marine fishes. Each point represents the mean niche with of all fish species with a 90% probability of occurring in that location. Species distribution was estimated from AquaMaps and habitat preference is obtained from FishBase.

The distribution of trophic position differed between habitats, primarily owing to the differences in the relative abundance of herbivorous fishes (i.e. trophic position of 2), which were absent in pelagic habitats but present in demersal and reef habitats (Fig. 1, Supporting Table 4). Latitudinal variation in the mean TP of species was evident across habitats whereby grid-squares close to the equator contained fishes foraging at a lower trophic position than those in polar seas (Fig. 4). A moderate negative relationship was evident between species’ trophic position and the number of times it occurred in the GBIF database (Table 1, Fig. 3), indicating that low trophic position fishes are more frequently observed. The marine productivity within a species geographic range and its dietary niche width were removed during model selection indicating that they had minimal influence on species trophic position.

**Table 1:** Results of generalized additive models (GAM) assessing global variation in dietary niche width (DNW) and trophic position (TP) of 2,938 marine fishes.

	Dietary Niche Width	Trophic position
n	2938	2938
r^2^	0.39	0.34
Dev explained (%)	39.4	34
Coefficient		
Productivity	3.2**	—
Biodiversity	79.4***	8.4***
SST range	3.9**	2.6*
Range	9.9***	7.4***
Length	44.4***	335.7***
GBIF occurrences	305.3***	21.9***
Trophic position	67.2***	NA
Dietary Niche Width	NA	46.8***
***Habitat***		
Bathydemersal	−0.09 (0.06)	0.27 (0.06)*
Bathypelagic	−0.26 (0.07)***	0.50 (0.09)***
Benthopelagic	−0.09 (0.06)	0.14 (0.06)*
Demersal	0.11 (0.03)***	0.07 (0.03)***
Pelagic - Neritic	−0.10 (0.07)	0.06 (0.07)
Pelagic - Oceanic	−0.32 (0.09)***	0.09 (0.10)
Reef-associated	0.04 (0.03)	−0.19 (0.03)***

Number of species (n), fit of each model (r^2^), percentage of deviance explained by each model in addition to, the coefficient of variation (equivalent to effect size as all variables were standardized) associated with each predictor variable.

*P < 0.05; **P < 0.01; ***P < 0.001; - removed during model selection; NA not included in model.

#### H1 – Relationship between richness and consumer trophic ecology

GAM’s revealed a negative relationship between a species’ DNW and the species richness within its geographic range (Fig. 3, Table 1). This trend was also evident in all habitats (Supporting Fig. S3, Table 1). Trophic position displayed a unimodal relationship with richness, whereby species in high and low richness regions were more likely to forage at a slightly lower trophic position that those in regions of moderate richness (Fig. 4, Table 1).

#### H2 – Species length and trophic ecology

The data supported our hypothesis that species size has a significant relationship with its trophic ecology. Fish length was positively related to both DNW and trophic position. Indeed, species length was, by some distance, the strongest predictor of trophic position (Fig. 3, Table 1) and this relationship was evident across all habitat types (Supporting Fig. S4).

#### H3 – Species range size and trophic ecology

The size of a species geographic range displayed a weak, positive relationship with DNW (Fig. 3). A stronger, positive relationship was observed between species range size and trophic position (Fig. 3, Table 1). This indicates that carnivorous or generalist species are likely to have a larger distribution than low trophic position or dietary specialists.

#### H4 – Relationship between species dietary niche width and trophic position

The hypothesized relationship between species DNW and trophic position was also evident in the data. Species with an intermediate trophic position had higher DNW than either obligate herbivores or piscivores (Table 1, Fig. 3). This trend was characteristic of most habitats, although it is difficult to ascertain its strength in pelagic habitats due to the absence of herbivorous taxa (Supporting Fig. S3).

## Discussion

Our results, based on an analysis of 2,938 fishes across seven major marine habitat types provide a novel insight into the relationship between consumer trophic ecology and species richness on a global scale, and support the current paradigm that competition for resources is an important driver of DNW in fishes, while also demonstrating the importance of species length and trophic position to this relationship. Species found in highly biodiverse regions, particularly reef-associated fishes, predominantly utilised a small DNW and foraged at a low trophic position. When considered in conjunction with the negative relationship between water temperature and species dietary niche width (Fig. S2), these results present a strong argument that fish communities in the warmest, most speciose seas on Earth are dominated by dietary specialists while consumers in the cooler, less biodiverse parts of the world typically employ a wider dietary niche width, and forage across multiple trophic positions.

These trends were evident in all habitat types, albeit with some significant differences. Most notably, no species with a trophic position <3, i.e. species which include primary producers in their diet, were observed in bathydemersal, bathypelagic and pelagic-oceanic habitats. Their absence likely reflects the paucity of macroalgae as a food item in pelagic habitats but also directly influences our ability to observe the predicted relationship between trophic position and dietary niche width in these regions. Similarly, species traits such as maximum size and range size are not evenly distributed between the habitats studied^9^ which may influence our ability to effectively model their relative importance in distinct habitats. For example, habitats in which length was not selected as an important predictor of dietary niche width (i.e. bathydemersal, benthopelagic and pelagic-neritic) contained a smaller range of maximum lengths than the habitats in which it was important. As such, it is plausible that the positive relationship between maximum length and dietary niche width evident in our model encompassing all fishes is also characteristic of these habitats, although this hypothesis requires validation.

The number of occurrences of a species in the GBIF database had a strong relationship with both metrics; it was the best predictor of a species dietary niche width and the third-best predictor, after species length and dietary niche width, of trophic position. Interpreting this relationship is challenging as it is not an ecological trait which could *a priori* be assumed to influence a consumer’s ecology. Our calculation of dietary niche width, integrating data from multiple sources, restricted our ability to assess the number of individual datapoints upon which our estimate of diet of any one species was made. As this metric of niche width is cumulative, the availability of more datapoints can increase but not decrease the number of prey items recorded in the diet of a species, and therefore its dietary niche width. Although the number of records of a species within the GBIF database is entirely independent from the number of datapoints upon which our estimate of diet was created, it is reasonable to assume that they would be correlated, i.e., that many diet data would be available for frequently observed species and less data available for less frequently observed species. The strong positive relationship between a species dietary niche width and the number of occurrences in GBIF supports this assumption and effectively partitions this source of variation within the model. As a result, the modelled relationships between dietary niche width and the other ecological traits measured (species richness, length, etc.) reflect variation in dietary niche width ‘corrected’ for the influence of sample size.

The negative relationship between number of occurrences of a species in GBIF and its trophic position likely has a different explanation. Trophic position is not a cumulative measurement. The observation of additional prey items in a species diet due to the presence of addition samples may cause the estimate of trophic position to increase or to decrease. Therefore, if we assume that the number of occurrences of a species in GBIF is broadly reflective of its abundance in nature we can interpret the negative relationship here as an indication that there are more low-trophic position than high-trophic position fishes in the Earth’s oceans. This explanation is commensurate with many similar scale studies of global fish community traits^7,10,11^.

No single environmental or biological predictor, with the possible exception of length on trophic position, explained an overwhelming about of the variation in either metric; species richness, trophic position and length each explained a similar proportion of dietary niche width in the near 3,000 fishes analyzed. The relationship between species richness and consumer dietary niche width provides insights into relationship between trophic ecology and ecosystem function. Current theory suggests that functional networks of species are the pre-eminent drivers of ecosystem function^29^. Thus, understanding how a species’ dietary niche width relates to its functional role within an ecosystem can help to shed light on the functional effects of biodiversity loss^30^. For example, increasing complexity of species interactions serves to mitigate the effect of biodiversity loss on ecosystem function^31^. In contrast, our findings suggest that as species richness decreases multiple specialists are replaced by fewer generalists, mitigating the loss of functional diversity while also limiting functional redundancy as multiple functional roles are filled by a smaller number of species^32^.

The findings in relation to trophic position and species length support our predictions that both of these would have a strong effect on consumer dietary niche width. Though length and trophic position are highly colinear their relationship to consumer dietary niche width differs: in contract to the linear positive relationship between dietary niche width and fish length, dietary niche width displayed a non-linear relationship with trophic position, whereby fish with an intermediate trophic position had a larger dietary niche width than specialist herbivores or piscivores. Fish are gape limited consumers, i.e. the can only feed on prey which fit in their mouth, and as such, our finding that lager fish feed on a greater variety of prey types to smaller fish is not surprising. Furthermore, large bodied fish have likely undergone several ontogenetic shifts during their life, with different life stages feeding on plankton, invertebrates and other fishes. They would be characterized as large bodied generalists in our DNW metric, though in reality each life stage may forage as a specialist. Our data reveal a general pattern whereby small bodied fishes predominantly forage as low trophic position specialists, intermediate sized fishes with a trophic position of 3–4 have a broad niche width and the largest bodied, highest trophic position fishes are specialist piscivores.

This study was limited to fishes, but we expect that the trends observed here would be evident in other groups. Studies of resource partitioning have shown similar relationships between specialists and generalists in reptiles, birds and mammals^33^, and there is strong evidence that resource diversity is linked to functional diversity in birds^34^ and insects^32^. We limited this work to fishes primarily because of the abundance of available data, e.g. NOAA^35^, ICES^36^, FishBase^8^ & GoMexSi^37^. The development of similar data archives for other groups would facilitate the extension of our analyses to include terrestrial consumers^38^.

Despite the clear picture presented here and its implications for wider studies, it is important to acknowledge certain biases, which may be associated with our data^39^. As the mined diet data were predominantly in the form of presence/absence we were unable to define the relative proportions of prey categories to the diet of each species and therefore were forced to include the implausible assumption that all prey categories were consumed equally when estimating dietary niche width. In addition, there is a significant mismatch between the scale at which we characterize richness (marine grid-square) and the scale at which a consumer’s dietary niche width is determined (i.e. habitat or site specific). We aimed to minimize these biases by developing a broad classification of dietary niche width (presence/absence of class level prey across the entire range of a species), and assessing variation on a global scale across multiple habitats. However, it is important to note that the trends reported here are associated with low r^2^ values indicating that a lot of variation within the dataset remains unexplained. It is also worth noting that the species included in our analysis represent a small proportion of the c. 12,000 marine fishes known to science^24^, and are biased towards well studied, commercially important Osteichthyes for which data are readily available. As these species are predominantly large and forage at a high trophic position it is likely that our estimates of mean dietary niche width and trophic position across the globe will need to be revised downwards as additional data become available. Commensurate with this, the relationships observed between trophic, environmental and biological traits are also biased towards large species. Our results indicate that small bodied fishes have a lower niche width than large fishes, a result that will likely be strengthened as further data become available.

A further limitation in our data is the collinearity between sea surface temperature and species richness. This relationship matches observations by Tittensor *et al*.^9^ and Stuart-Smith *et al*.^7^ that marine biodiversity is positively correlated with sea surface temperature but poses a challenge to our interpretation of the relationship between species richness and trophic ecology. As both variables are colinear, our model could not discern whether variation in trophic ecology was due to the water temperature or species richness within a fish’s range. We therefore omitted sea surface temperature from our models as, at a functional level, a consumer’s trophic ecology is primarily determined by resource availability^40^. However, as fish are poikilotherms their foraging behavior is influenced by ambient water temperature, species inhabiting cold water environments will be characterized by lower metabolic rate which may influence the frequency at which they feed and the breadth of prey items they are exposed to^41^. Determining such physiological drivers of consumer trophic ecology is beyond the scope of this dataset but represents an area ripe for further investigation. Based on our findings, it is reasonable to conclude that consumers in the warmest most species-rich parts of the ocean are likely to have a smaller dietary niche width that those in cooler less-diverse regions.

This work adds to a growing body of material using consumer’s functional traits to develop a broader understanding of ecosystem function^7,42^. For example, Micheli & Halpern^43^ detail a strong correlation between species richness and functional diversity in marine fishes. This is commensurate with our findings that fishes in diversity hotspots are likely to be small dietary specialists foraging at a low trophic position. Similarly, our data indicate that fishes in polar seas have a larger dietary niche than those in equatorial regions, supporting recent work by van Denderen *et al*.^25^ who show that piscivores in equatorial regions predominantly forage on pelagic prey whereas those foraging in polar seas feed on both benthic and pelagic fishes. Beyond the aquatic biome, a similar approach has been used to characterize the evolution of functional diversity in mammals^44^. The increased availability of trait based data archives for a wide diversity of biota represents an opportunity to apply the approach to some of the foundational theories of ecology^38,42,45^.

In conclusion, dietary niche width is determined by a variety of biological and environmental traits but, at a global level, is predominantly related to the prey and competitor species richness within a consumers range. Dietary niche width is however, just one component of a species ecological niche and future investigations examining the macro-ecological relationship between species richness and ecological niche width metrics^46^ would be revealing in this regard.

## Supplementary information

Supporting Information

Supporting Table 2
