# Supplementary material for: Biological and environmental drivers of trophic ecology in marine fishes - a global perspective
Source: Sci Rep. 2019 Aug 6;9:11415. doi: 10.1038/s41598-019-47618-2 (PMC6684618; doi:10.1038/s41598-019-47618-2)

A global assessment of the biological and environmental drivers of trophic ecology in marine fishes

B Hayden^1 *^, MLD Palomares^2,3^, BE Smith^4^. and JH Poelen^5^.

**Supporting Information**

**Supporting Table 1.** List of all data sources from with diet information was mined. Database export shared by http://fishbase.org in December 2013.

1. <http://www.hindawi.com/dpis/ecology/2013/857470/dataset/>
2. Thessen. 2014. Species associations extracted from EOL text data objects via text mining. Accessed at https://raw.github.com/EOL/pseudonitzchia/master/associations_all_revised.txt on 14 Nov 2014.
3. Semantic Prototypes in Research Ecoinformatics (SPIRE). Data provided by Joel Sachs. See also http://ebiquity.umbc.edu/get/a/publication/297.pdf .
4. Southern California Bight Kelp Forest Food Web data provided by Jarrett Byrnes. Also available at http://dx.doi.org/10.1111/j.1365-2486.2011.02409.x
5. Barnes, C. et al., 2008. PREDATOR AND PREY BODY SIZES IN MARINE FOOD WEBS. Ecology, 89(3), pp.881â€“881. Available at: http://dx.doi.org/10.1890/07-1551.1 . Data provided by Carolyn Barnes. Also available at http://www.esapubs.org/Archive/ecol/E089/051/ .
6. International Council for the Exploration of the Sea. Available at http://www.ices.dk/products/cooperative.asp .
7. Brose, U. et al., 2005. Body sizes of consumers and their resources. Ecology 86:2545. Available from doi:10.1890/05-0379 .
8. http://gomexsi.tamucc.edu
9. http://www.nefsc.noaa.gov/femad/pbio/fwdp/
10. Benjamin Planque, Raul Primicerio, Kathrine Michalsen, Michaela Aschan, GrÃ©goire Certain, Padmini Dalpadado, Harald GjÃ¸sÃ_ater, Cecilie Hansen, Edda Johannesen, Lis Lindal JÃ¸rgensen, Ina Kolsum, Susanne Kortsch, Lise-Marie Leclerc, Lena Omli, Mette Skern-Mauritzen, and Magnus Wiedmann 2014. Who eats whom in the Barents Sea: a food web topology from plankton to whales. Ecology 95:1430â€“1430. http://dx.doi.org/10.1890/13-1062.1
11. Raymond, B., Marshall, M., Nevitt, G., Gillies, C., van den Hoff, J., Stark, J.S., Losekoot, M., Woehler, E.J., and Constable, A.J. (2011) A Southern Ocean dietary database. Ecology 92(5):1188. Available from http://dx.doi.org/10.1890/10-1907.1 . Data set supplied by Ben Raymond. Accessed at https://www1.data.antarctica.gov.au/aadc/trophic/trophic.zip on 14 Nov 2014.
12. http://www.esapubs.org/archive/ecol/E092/066/
13. http://iNaturalist.org is a place where you can record what you see in nature, meet other nature lovers, and learn about the natural world. Accessed at http://inaturalist.org on 14 Nov 2014.
14. Data provided by Colt W. Cook. Also available from http://repositories.lib.utexas.edu/handle/2152/ETD-UT-2012-08-6285.
15. Giovanni Strona, Maria Lourdes D. Palomares, Nicolas Bailly, Paolo Galli, and Kevin D. Lafferty. 2013. Host range, host ecology, and distribution of more than 11800 fish parasite species. Ecology 94:544. http://dx.doi.org/10.1890/12-1419.1 . Accessed at http://www.esapubs.org/archive/ecol/E094/045/FPEDB.csv on 13 Nov 2014.
16. Polytraits, a database on biological traits of polychaetes. Available at http://polytraits.lifewatchgreece.eu.
17. Food Webs and Species Interactions in the Biodiversity of UK and Ireland (Online). 2013. Data provided by Malcolm Storey. Also available from <http://bioinfo.org.uk>.

**Supporting Table 2.** Diet table listing the presence/absence of 29 prey categories in the recorded diet of all fishes used in the presented analysis. The values dietary niche width (DNW) and trophic position (TP) of each species and the predictor variables used in all models are also provided. <https://www.dropbox.com/s/egth4kl2cubkczi/Table%20S2.csv?dl=0>

**Supporting Table S3.** Pairwise t-test comparisons of variation in dietary niche width of fishes between habitats. Values represent Bonferroni corrected P values. Statistically significant (alpha < 0.05) values are highlighted in bold

bathydemersal bathypelagic benthopelagic demersal pelagic-neritic pelagic-oceanic

bathypelagic 0.867 - - - - -

benthopelagic 1.000 0.054 - - - -

demersal 1.000 **<0.01**  1.000 - - -

pelagic-neritic 0.822 1.000 0.081 **<0.01**  - -

pelagic-oceanic 1.000 1.000 1.000 1.000 1.000 -

reef-associated **<0.01 <0.05 <0.01 <0.01**  1.000 **<0.01**

**Supporting Table S4.** Pairwise t-test comparisons of variation in trophic level of fishes between habitats. Values represent Bonferroni corrected P values. Statistically significant (alpha < 0.05) values are highlighted in bold

bathydemersal bathypelagic benthopelagic demersal pelagic-neritic pelagic-oceanic

bathypelagic 0.254 - - - - -

benthopelagic 1.000 1.000 - - - -

demersal **<0.01** **<0.05**  **<0.01**  - - -

pelagic-neritic **<0.01**  0.401 0.088 1.000 - -

pelagic-oceanic 1.000 0.061 0.361 **<0.01**  **<0.01**  -

reef-associated **<0.01**  **<0.01** **<0.01** **<0.01**  **<0.01** **<0.01**

**Supporting Figure S1.**  GAM smoothers detailing the relationship between predictor variables dietary niche width (DNW) and trophic level (TL) of fish measured as (a) total length (n=1828) and (b) standard length (n=667).

**Supporting Figure S2.**  Pairs plots outlining the ‘raw data’ relationship between trophic traits and predicted correlates. Scatter plots, density distributions and correlation coefficients are provided for each combination of variables.


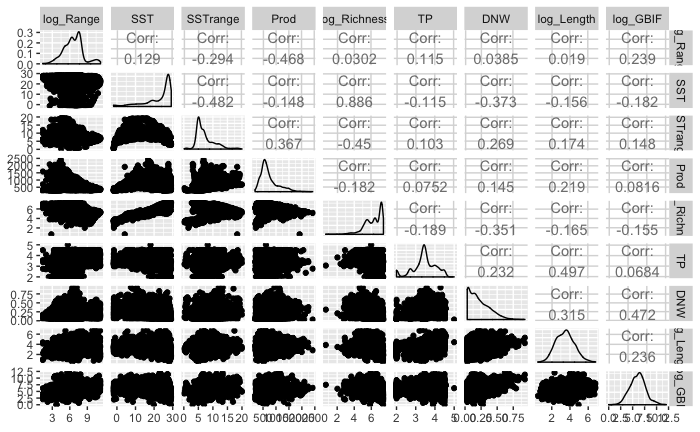


**Figure S3.** Relationship between dietary niche width and predictor variables of 2,580 fishes across major marine habitat types. Plots represent relationships indicated by the best fitting GAM (see Table 1). LOESS smoothers are fitted to each dataset, shading denotes 2 standard errors.


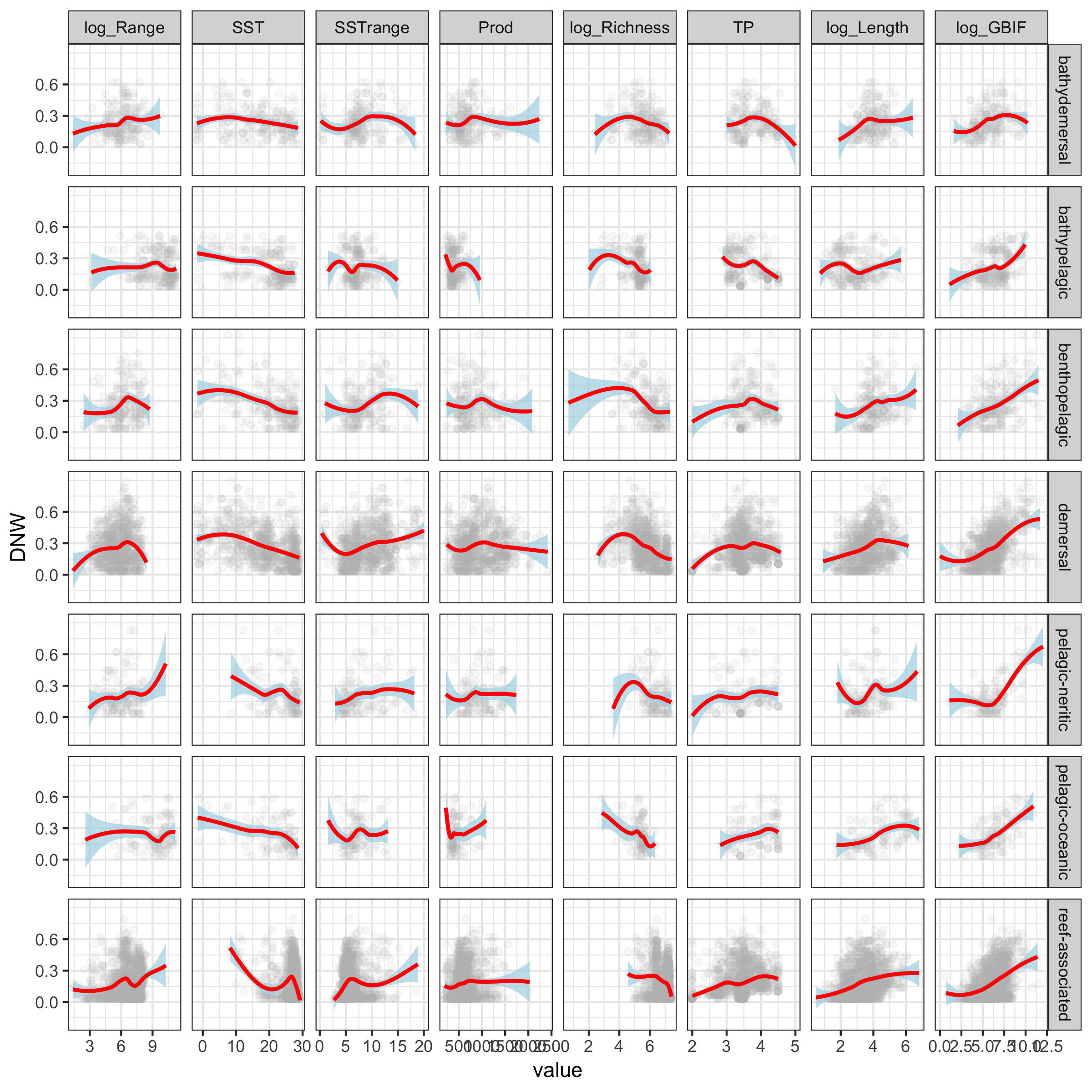


**Figure S4.** Relationship between trophic level and predictor variables of 2,580 fishes across major marine habitat types. Plots represent relationships indicated by the best fitting GAM (see Table 1). LOESS smoothers are fitted to each dataset, shading denotes 2 standard errors.


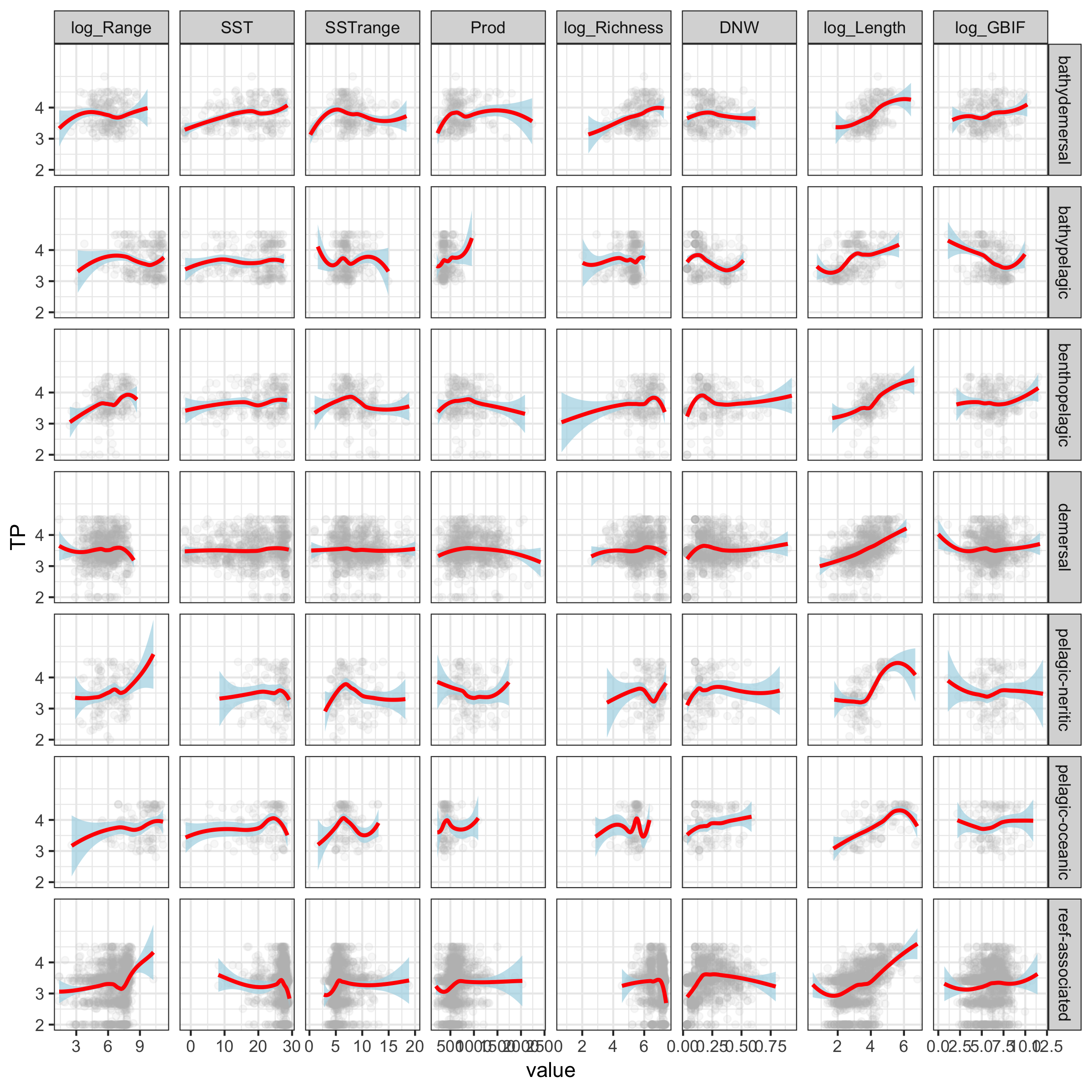

Supplement: Supplementary file 1 — Supporting Information [file 41598_2019_47618_MOESM1_ESM.docx]
